# Lipid Specific Membrane Interaction of Aptamers and Cytotoxicity

**DOI:** 10.3390/membranes12010037

**Published:** 2021-12-27

**Authors:** Md. Ashrafuzzaman, Hanouf A. M. AlMansour, Maha A. S. AlOtaibi, Zahid Khan, Gouse M. Shaik

**Affiliations:** Biochemistry Department, Science College, King Saud University, Riyadh 11451, Saudi Arabia; 439204012@student.ksu.edu.sa (H.A.M.A.); 439203105@student.ksu.edu.sa (M.A.S.A.); zahidatkhan@yahoo.com (Z.K.); gshaik@KSU.EDU.SA (G.M.S.)

**Keywords:** aptamer, phosphatidylserine, membrane, cell, cytotoxicity

## Abstract

We aim to discover diagnostic tools to detect phosphatidylserine (PS) externalization on apoptotic cell surface using PS binding aptamers, AAAGAC and TAAAGA, and hence to understand chemotherapy drug efficacy when inducing apoptosis into cancer cells. The entropic fragment-based approach designed aptamers have been investigated to inspect three aspects: lipid specificity in aptamers’ membrane binding and bilayer physical properties-induced regulation of binding mechanisms, the apoptosis-induced cancer cell surface binding of aptamers, and the aptamer-induced cytotoxicity. The liposome binding assays show preferred membrane binding of aptamers due to presence of PS in predominantly phosphatidylcholine-contained liposomes. Two membrane stiffness reducing amphiphiles triton X-100 and capsaicin were found to enhance membrane’s aptamer adsorption suggesting that bilayer physical properties influence membrane’s adsorption of drugs. Microscopic images of fluorescence-tagged aptamer treated LoVo cells show strong fluorescence intensity only if apoptosis is induced. Aptamers find enhanced PS molecules to bind with on the surface of apoptotic over nonapoptotic cells. In cytotoxicity experiments, TAAAGA (over poor PS binding aptamer CAGAAAAAAAC) was found cytotoxic towards RBL cells due to perhaps binding with nonapoptotic externalized PS randomly and thus slowly breaching plasma membrane integrity. In these three experimental investigations, we found aptamers to act on membranes at comparable concentrations and specifically with PS binding manner. Earlier, we reported the origins of actions through molecular mechanism studies—aptamers interact with lipids using mainly charge-based interactions. Lipids and aptamers hold distinguishable charge properties, and hence, lipid–aptamer association follows distinguishable energetics due to electrostatic and van der Waals interactions. We discover that our PS binding aptamers, due to lipid-specific interactions, appear as diagnostic tools capable of detecting drug-induced apoptosis in cancer cells.

## 1. Introduction

In drug discovery and biosensor development, aptamers have been found to create popular classes of agents [1,2,3,4] since the first breakthrough development of a novel aptamer discovery method, ‘systematic evolution of ligands by exponential enrichment (SELEX)’ by Tuerk and Gold in 1990 [5]. We are part of teams having developed two novel methods, the ‘entropic fragment-based approach (EFBA)’ [6,7] and the energy-based method utilizing screened Coulomb interactions (EBM-SCI) [8], to design aptamers applying sets of theoretical and computational methods and experimental validation techniques. Aptamers are oligonucleotide or peptide molecules having the potency to bind specifically to target biomolecules. Our group has recently discovered a few nucleic acid aptamers (NAAs) using EFBA to specifically bind to phosphatidylserine (PS), a targeting lipid which is an important biomarker to diagnose induction of apoptosis in cancer cells, so these aptamers (let us call them ‘PS aptamers’) are considered diagnostic agents [6,7]. Both EFBA and EBM-SCI have been found to design both diagnostic [6,7] and therapeutic (manuscript in preparation, Ashrafuzzaman) aptamers to deal with cancer and other diseases subject to the availability of information about the target biomolecule(s) and the environment hosting the target biomolecules (US patent, pending by Ashrafuzzaman). In this article, we elaborate our understanding on aptamers’ target PS binding potency by presenting liposome and cancer cell binding experimental data, thus demonstrating the PS binding of aptamers in both controlled environment and biological systems using three independent experimental techniques, namely, in vitro liposome binding assays, cell surface fluorescence imaging, and cytotoxicity measurements.

Phospholipids are among the major components constructing the cellular membrane [9]. They play crucial roles in maintaining cellular structures and versatile functions. Different drugs including ones used as chemotherapy drugs (CDs) are often found to target cell membrane constituents [10,11]. General cell surface binding and specific lipid interactions have recently been claimed as hallmark mechanisms for CD cytotoxicity [12,13,14]. Here, we have found that CD molecular interactions with cell membranes happen mainly due to physical drug–lipid interactions, leading to physical drug clustering on the cell surfaces, which further causes drug distribution across the membrane and creates drug type-specific ion pores [12]. Lipid targeted drug actions to induce apoptosis in cancer cells is now an accepted hypothesis [15]. Here an alkyl-lysophospholipid analog edelfosine was found as an antitumor drug capable of inducing apoptosis via co-clustering of lipid rafts and the Fas/CD95 death receptor. All these background studies suggest that the cell membrane in general and the membrane’s certain lipids specifically may play crucial roles while experiencing the presence of CDs in membranes’ hydrophobic core or on the surface. We know in eukaryotic cells, major membrane phospholipid components are found to be the glycerophospholipids phosphatidylcholine (PC), the phosphatidylserine (PS), the phosphatidylethanolamine (PE), the phosphatidylinositol (PI), and the phosphatidic acid (PA) [16]. Over about two billion years, cells have got their maturity having functional cell membranes that comprise a clear composition of various lipid components, domains, etc. PC represents more than 50% of phospholipids. These molecules bear no net charges, so they function as neutral type lipids, responsible for contributing to creating planar lipid bilayers. In contrast, PS is known to exist in relatively low concentrations, ~10% of the membrane lipids, playing crucial roles in cellular processes [17,18]. PS molecules are synthesized in the mammalian cell through base-exchange reactions via the replacement of polar head groups of preexisting phospholipids by serine. The PS level is maintained or regulated by multiple compensatory cellular mechanisms. 

PS is naturally involved in regular cellular events such as apoptosis and other cell signalings [17]. Particularly, the involvement of PS with apoptosis is our interest in this study [6,7]. Migration of PS molecules or PS externalization across the plasma membrane towards the extracellular surface is one of the major hallmarks of apoptosis [19,20,21,22,23]. Early-stage detection of the apoptosis using a fluorescent conjugate of annexin V (annexin A5) was made quite some time ago, e.g., see Reference [24]. Here, the translocation of PS molecules from the inner face of the plasma membrane to the cell surface was detected. Annexin V, a naturally occurring human PS-binding protein, is usually investigated as either a radionuclide containing or any fluorescent probe to detect the PS externalization [25]. However, annexins exhibit various disadvantages including the high uptake in normal tissues, the long half-life in non-target tissues, the high level of the radiation burden with radiolabeled tracers, and the laborious labeling [26]. We are therefore in search of an alternative. We found a method EFBA and designed NAAs that would detect the migrated PS on the cell surface [6,7]. The aptamers were experimentally found to prefer binding with their target lipids over other ones in liposomes [6,7]. That says the EFBA designed aptamers are lipid specific, which is usual as in the design technique they are made to bear physical characteristics favoring the specific target(s) in the right biological environments. We already demonstrated these matters in our earlier publications [6,7] and selected two PS aptamers (PS binding aptamers: AAAGAC and TAAAGA), which should bind preferentially with PS molecules in lipid membranes. Besides liposome binding assays, we also applied molecular dynamics (MD) simulations to explore the possible aptamer preference to bind with specific lipids over other ones, considering mainly the aptamer–lipid interaction energetics [6,7,13]. In these articles, we clearly demonstrated that our discovered PS aptamers show preferred pairwise binding with PS molecules over other lipids considering their charge properties-based interactions. Both electrostatic and van der Waals interactions have been found to contribute to the binding energies. 

In the current article, we have planned to elaborate on our understanding of the PS aptamer binding with PS lipids in the biological environment. Before going to actual biological assays, we wished to address first how the bilayer environment regulates the lipid-specific aptamer binding with liposomes. We used two amphiphiles, triton X-100 (TX100) and capsaicin (Cpsn), which are known to alter bilayer physical properties [27,28], consequently regulating the function of membrane adsorbed agents, e.g., CD agent colchicine [29], antimicrobial peptides gramicidin A [28,30,31], and alamethicin (see Supplementary Materials of Reference [29]) inside lipid bilayers. Besides reducing bilayer stiffness (represented by reduced modulus of elasticity [27] which helps make the bilayer softer or more elastic) both TX100 and Cpsn are known to promote positive and negative lipid curvature profiles, respectively, in the lipid bilayer membrane. Therefore, both amphiphiles are predicted to also influence the physical membrane adsorption of aptamers, so we planned to check them here regarding their effects on membrane adsorption of aptamers. After this in vitro binding experiment, we focused on cell culture assays in which we performed two independent studies; firstly, whether we could image the target lipid-bound aptamers in the membrane. Then, we addressed another important aspect, cytotoxicity. Nonapoptotic PS externalization can be achieved by the engagement of glycosylphosphatidylinositol-anchored proteins [32]. Here, the engagement of GPI-APs in rodent mast cells has been found to induce a rapid and reversible externalization of PS by a nonapoptotic mechanism. Considerable shreds of evidence were also provided earlier that the IgE-dependent stimulation of rat mast cell lines, as well as murine and human non-transformed mast cells, leads to the exposure of PS at the plasma membrane, suggesting for PS externalization in mast cells to be not necessarily related to apoptosis but could be an important feature of the degranulation process [33]. It is well known that an increase in calcium influx increases PS externalization. We applied this strategy to ensure the PS externalization on nonapoptotic cells for cytotoxicity assays [34]. Checking the cytotoxicity of a drug on this kind of nonapoptotic cells may be a good choice because in the current study, we aim at checking the cytotoxicity potency of our PS aptamers due to a specific molecular mechanism—the PS molecule binding of drugs. Therefore, avoiding another rather complex cellular process (alongside achieving the PS binding of PS aptamers), induction of apoptosis may be a good choice. In this case, cells are expected to react to aptamer drugs through binding of PS aptamers with PS molecules in this kind of nonapoptotic cell condition [32]. Due to such target lipid binding in the membrane, whether these PS aptamers are found considerably cytotoxic was our objective to explore. 

We used aptamers that are PS molecule binding agents so that they could be characterized and medically used as apoptosis diagnostic tools, since apoptotic cells (over normal ones) appear with available PS molecules on cell surfaces. We used two of the designed PS aptamers SIAp3 (TAAAGA) and SIAp4 (AAAGAC) (see Supplementary Table S1 and Supplementary Figure S1). We also chose an aptamer SIIAp1 = CAGAAAAAAAC as a negative control (poor PS binding agent, see Supplementary Table S1). We picked these three agents to address their lipid-specific membrane binding potency and as a result their cytotoxicity. Both PS aptamers have been found to get liposome adsorbed substantially in a specific PS binding manner, and the bilayer physical property altering amphiphiles regulate their membrane adsorption process. Fluorescence images suggest the PS aptamers bind to cell surfaces as PS molecules pop up due to the induction of apoptosis into cancer cells. PS aptamers show modest cytotoxicity due to perhaps their binding to the target lipids in the cell membrane.

## 2. Materials and Methods

### 2.1. Liposome Binding Assays-FL Measurements on PS Bound Aptamers

Membrane binding effects of aptamers were measured using FL assays with aptamers bound to liposomes constructed using 1,2-dipalmitoyl-sn-glycero-3-phosphatidylcholine (DPPC), and 1,2-dipalmitoyl-sn-glycero-3-phospho-L-serine (sodium salt) (DPPS) (lipids were purchased from Avanti Polar Lipids, 700 Industrial Park Dr, Alabaster, AL, USA). 6-carboxyfluorescein (6-FAM), fluorescent tag, was attached to aptamer DNA’s 3’ end. We purchased lipids from Avanti Polar Lipids (700 Industrial Park Drive Alabaster, AL, USA) and aptamers from HVD Biotech Vertriebs GmbH (Wurzbachgasse 18, 1150, Vienna, Austria). Standard assays for binding were utilized (for details, see our previous publications [6,7] and Supplementary Materials ‘Aptamer-liposome binding assays’) to separate liposome-bound aptamers from unbound ones in the buffer. The liposome-bound aptamer solution was investigated to quantify for FL. The 96 well assay plates for fluorescence assays using FLUOstar OPTIMA (BMG LABTECH GmbH, Offenburg, Germany) were from Corning Inc. (New York, NY, USA). There were eight replicates of samples prepared for each aptamer concentration in the studies. A 2:1 ratio of PC to cholesterol was prepared by dissolving 100 mg of DPPC and 24.45 mg of cholesterol in 10 mL of chloroform to prepare control liposomes that do not contain PS. PS containing liposomes contained a combination of DPPC and DPPS in a 10 to 1 molar ratio. The pH of the aqueous buffer was maintained at 7.4.

To investigate the effects of amphiphiles on liposome adsorption of aptamers we added both TX100 or Cpsn stock and aptamers (binary mixture of amphiphile and aptamer) in the aqueous phase bathing the liposome (see ‘Aptamer-liposome binding assays’ in Supplementary Materials). The concentrations of TX100 and Cpsn used here are known, in earlier studies [27,28,29], not to alter the bilayer’s gross electrical insulation properties. We then repeated the previously explained method to separate liposome-bound aptamers from unbound ones in buffer and then performed the FL measurements on liposome-bound aptamer solution. At the least, we performed three experimental repeats so that we could demonstrate the statistical nature of the effects. TX100 and Cpsn were purchased from Sigma (Sigma-Aldrich Handels Gmbh, Marchettigasse 7/2, 1060 Wien, Austria).

### 2.2. Liposome Binding Assays-DDM to Measure Liposome Bound Aptamer Concentrations

A direct detection method (DDM) was applied in order to detect lipid-bound aptamers in mole (M) fraction [13,35]. Similar to the Section 2.1, to investigate the effects of amphiphiles on liposome adsorption of aptamers, we added both TX100 or Cpsn stock and aptamers (binary mixture) in the aqueous phase bathing the liposome. In our earlier publications, we already addressed DDM for detecting aptamers in vitro DPPC and DPPS liposome systems using standard absorbance spectroscopy (details in References [13,35]). DDM helps detect molecules directly at the target or binding site(s). Let us denote B and UB for the solutions separated as the mole fraction of aptamers bound to lipids and unbound ones, respectively. As mentioned earlier, the liposome bound and unbound aptamers in solution got separated, and hence, we obtained solutions B and UB, using methods explained in References [13,35] (we avoid repeating these published materials here). We then used a NanoDrop (purchased from ThermoFisher Scientific, Waltham, MA, USA) or a Nanophotometer (purchased from Implen GmBH, München, Germany) to get the absorbance spectra that are specific for certain aptamers. The wavelength (λ_MAA_) of the spectrum is actually membrane-active agent (MAA) specific. λ_DNA_ = 260 nm is for DNA aptamers (please see the Sigma-Aldrich manual). We then performed the spectroscopy on both samples and quantified the concentrations of aptamers dissolved in both samples B and UB [13,35]. Using these detected concentrations, we calculated the molarities of both the lipid-bound and lipid-unbound aptamers in the incubation tube. These concentrations were then normalized with the correct volume of the aqueous buffer in which the liposomes were formed, and then, the liposomes were incubated with drugs before splitting the whole solution into B and UB. The lipid-bound drugs are nothing but considered as the DDM detected liposome-bound drugs that are plotted later in this article. At the least, we performed three experimental repeats to demonstrate the statistical nature of the effects.

### 2.3. Cell Culture and Imaging Experiments

Cell culture and colchicine treatment. Human colorectal adenocarcinoma cells, LoVo, were maintained in Dulbecco’s modified Eagle’s medium (DMEM; Gibco, Waltham, MA, USA) supplemented with 10% fetal bovine serum (FBS; Gibco) and 50 units/mL penicillin-50 μg/mL streptomycin (Gibco). At 75–80% confluence, LoVo cells were treated with 2 µM colchicine (from Sigma, St. Louis, MO, USA) for 24 h to induce apoptosis. Colchicine is known to induce apoptosis into cancer cells [36,37]. Cells were treated with 100 µM aptamer (aliquot taken from 10 mM stock in DMSO) for 1 h or Annexin V Alexa Fluor 488 conjugate. Aptamers were conjugated to FAM fluorescent dye, and annexin V was conjugated to Alexa fluor 488. Experiments with annexin V were performed for comparing the aptamer data. Cells were counted using a handheld automated cell counter, Scepter (Millipore, Burlington, MA, USA) fitted with 60 μm sensors to a final concentration of 5 × 10^5^ cells/mL in PBS for microscopic imaging investigations using fluorescence microscope IX51 (Olympus, Tokyo, Japan).

### 2.4. Cell Culture and Cytotoxicity Experiments

**Cell culture.** Sample of RBL cells was obtained with thanks from the Signal transduction department of Institute of Molecular Genetics, Academy of Science of Czech Republic, Prague. Cells were maintained in Dulbecco’s modified eagle’s essential medium (DMEM) supplemented with 10% FBS. Cells were passaged every 48 h with the help of 0.25% trypsin EDTA solution. Cells were incubated at 37 °C in a humidified chamber with 5% CO_2_. Cells were seeded in a 96 well cell culture plate 24 h before the actual experiment. For activation of cells, calcium ionophore A23187 (Sigma Aldrich C7522) was employed at 1 µM final concentration following the method explained in Reference [32]. This way we increased the intracellular calcium level which results in the PS externalization [34]. The stock solution of control aptamer SIIAp1 and PS aptamer SIAp3 was diluted in DMEM to the required final concentrations and incubated for 24 h. To record morphology, pictures of cells were taken using a Leica microscope DFC450 equipped (Leica Camera AG, Wetzlar, Germany) with a camera at 20× magnification.

**Cytotoxicity assay.** To assess the cytotoxic effect of the selected PS aptamer, we performed 3-(4,5-dimethylthiazol-2-yl)- 2,5-diphenyltetrazolium bromide dye reduction assay (MTT assay) [38]. Briefly, RBL cells were counted and 10,000 cells seeded in a 96 well flat bottom culture plate. After 24 h, cells were briefly activated by 1 µM calcium ionophore A23187 and subsequently treated with 100 µM control aptamer or PS aptamer (1, 10, and 100 µM). 

Treated cultures were incubated for 24 h at 37 °C in a humidified incubator with 5% CO_2_. After incubation, 10 µL of MTT stock (5 mg/mL in phosphate buffered saline) was added to the cultures and further incubated for 4 h. At the end of the incubation, 100 µL of dimethyl sulfoxide was used to dissolve the formed formazan. Absorbance was measured using an ELISA plate reader (Spectra MAX Molecular devices, Molecular Devices, San Jose, CA, USA) with a 460 nm setting. Normalized data from 3 independent experiments are reported.

## 3. Results

We have performed three independent sets of experiments to address the membrane adsorption of PS aptamers and associated cytotoxicity. Firstly, the lipid specificity in aptamer binding with the membrane and the regulation of the membrane-binding mechanisms due to alterations in bilayer physical properties have been addressed using in vitro liposome binding assay experiments. Secondly, the cell surface binding of PS aptamers while cancer cells are induced with apoptosis has been addressed using imaging experiments. Thirdly, we inspected the cytotoxicity induced by PS aptamers on cancer cell lines. 

### 3.1. Amphiphiles Regulate the Liposome Adsorption of PS Aptamers

Liposome adsorption of aptamers has already been demonstrated in our earlier published articles using two independent methods, FL measurements [6,7] and DDM [13,35]. For our PS aptamers, we found that the liposome binding of these aptamers happens using PS specific interactions. If the liposome contained no PS molecules, negligible liposome binding of aptamers was reported, see Supplementary Figure S1 [7]. We wish to see here if bilayer physical properties regulating amphiphiles [27,28] may also influence the mechanisms of the membrane adsorption of aptamers. 

**Figure 1:**
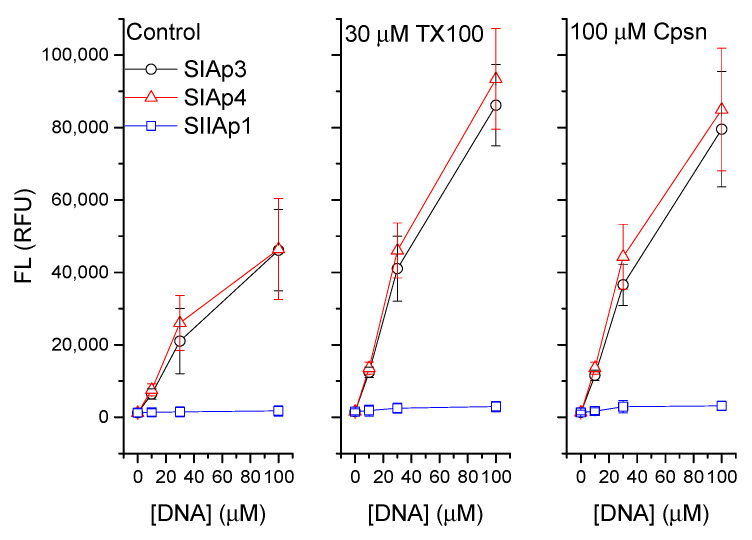
Measured FL on the liposome bound aptamers are plotted against various concentrations of aptamers added to the aqueous buffer bathing the liposome (**left figure**). Middle and right figures represent the same as the left figure except for the cases where 30 μM TX100 and 100 μM Cpsn, respectively, were added concomitantly with aptamers in the buffer. Here, we have used the two best PS aptamers, SIAp3 (TAAAGA) and SIAp4 (AAAGAC), and an insignificant PS binding aptamer, SIIAp1 (CAGAAAAAAAC) (see Supplementary Table S1). Quantitative liposome binding (represented through measured FL on the bound aptamers, left panel) of both aptamers generally increases due to effects of both TX100 (**middle panel**) and Cpsn (**right panel**).

**Figure 2:**
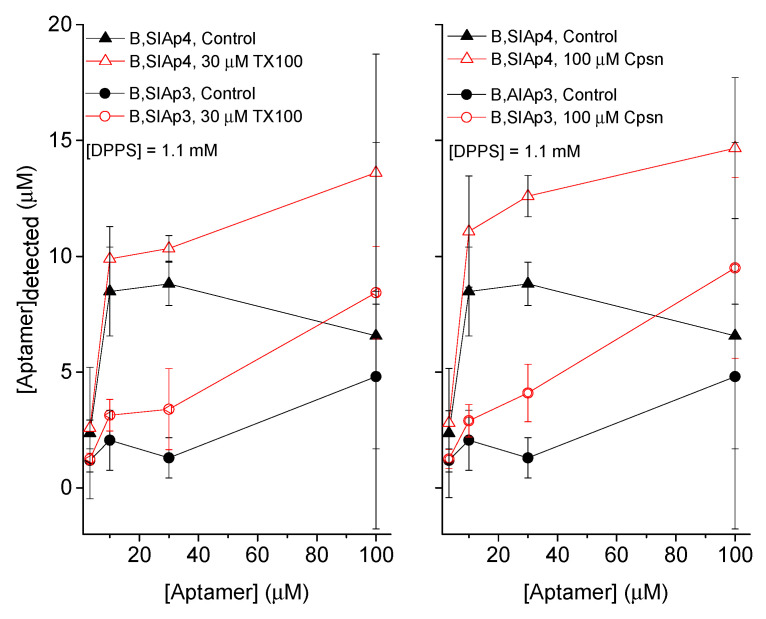
DDM detection of PS bound ‘PS aptamers’ (SIAp3 and SIAp4), measured directly at the liposomes. Quantitatively, we find here that the liposome binding of both aptamers generally increases due to both TX100 (**left panel**) and Cpsn (**right panel**). [DPPS] = 1.1 mM was the lipid concentration in the aqueous phase.

In Reference [13], we observed only about 10% aptamer binding to PS liposome, but it was negligible with PC liposomes (data are not shown here). We chose here only a few low aptamer concentrations for testing whether their PS liposome binding potency gets influenced due to the amphiphile effects on lipid membranes. We considered 30 μM TX100 and 100 μM Cpsn; both agents at these concentrations are well known to influence the bilayer stiffness by most likely increasing the bilayer elasticity [27,28]. This amount of amphiphiles, while concomitantly added with aptamers in the aqueous buffer that incubates liposomes, has clearly been found quantitatively to increase the membrane adsorption of aptamers. This is reflected in the increased fluorescence (Figure 1) and increased detected aptamers in the liposomes (Figure 2).

### 3.2. Imaging Experiments Tracking PS Aptamers on the Cell Surface

**Figure 3:**
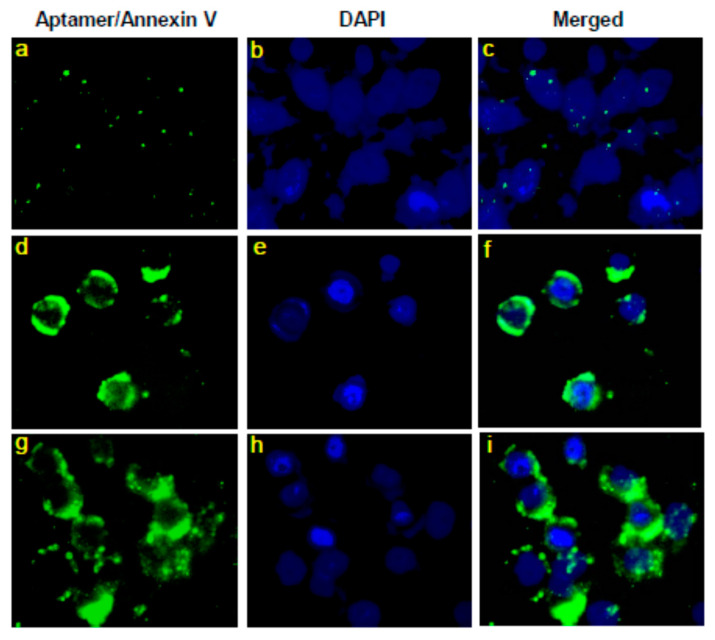
Aptamer binding to apoptotic cells. LoVo cells were treated with 2 µM colchicine for 24 h to induce apoptosis (**d**–**i**). (**a**–**c**) Control cells without colchicine treatment. Cells were treated with 100 µM aptamer SIAp3 (TAAAGA) for 1 h (**a**–**f**) or Annexin V Alexa Fluor 488 conjugate (**g**–**i**). Aptamers were conjugated to FAM fluorescent dye (**a**,**d**) and annexin V was conjugated to Alexa fluor 488 (**g**). The fluorescent stain DAPI (4′,6-diamidino-2-phenylindole) images (**b**,**e**,**h**) show no presence of fluorescence like that observed from aptamer SIAp3 or Annexin V (left column). Merging the left and middle columns shows the combined colors with clear contrast in images (**c**,**f**,**i**). 400× magnification.

A representative fluorescence microscopic image depicting the cancer cell line (with induced apoptosis) binding of our diagnostic PS aptamers is presented here. Comparison between Figure 3a and Figure 3d or Figure 3c and Figure 3f suggests that due to induction of apoptosis by colchicine [36,37], PS molecules migrate through the apoptotic process to the surface of the cell, and thus, more PS aptamers are found (represented by a higher intensity of fluorescence) in their PS bound state (see Figure 3d) than negligible fluorescence observed in Figure 3a. The condition represented by Figure 3a with poor fluorescence intensity does not suggest favoring the possibility of the aptamers cell internalization, as then, we would find huge fluorescence intensity due to binding of internalized aptamers with PS molecules on the intracellular surface of the membrane. The other agent, Annexin V, has been used to compare our aptamer results with those images as those are naturally used for this kind of imaging experiment. Therefore, our proof of principle on imaging the FAM tagged PS aptamers is found to work, demonstrating the lipid specificity in aptamer binding on the cell surface.

### 3.3. PS Aptamer-Induced Cytotoxicity Results

**Figure 4:**
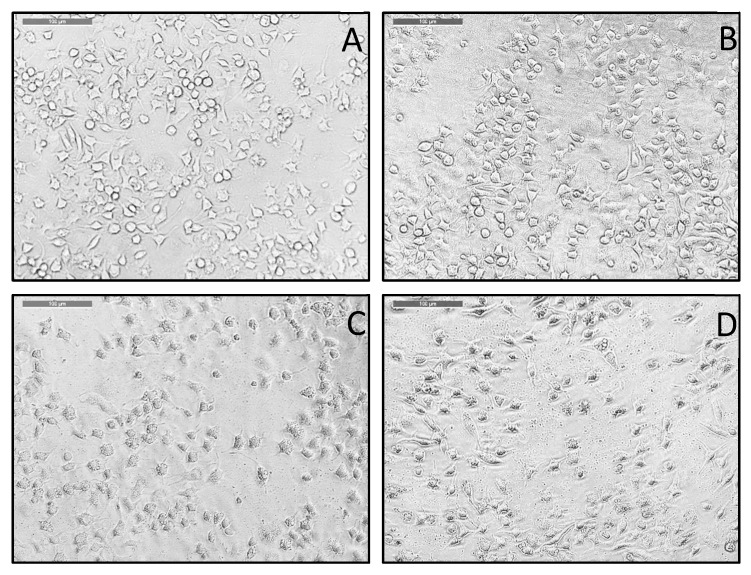
PS aptamer treatment induces morphological changes in RBL cells. Calcium ionophore activated RBL cells treated with 100 µM control or nonspecific aptamer SIIAp1 (CAGAAAAAAAC) (**A**) or with PS aptamer SIAp3 (TAAAGA) 1 µM (**B**), 10 µM (**C**), and 100 µM (**D**). Cells showing morphological changes with the increase in PS aptamer concentration. At concentration 100 µM (**D**), increased granulation is observed as evidenced by dark cytoplasm.

In our studies, when RBL cells were activated by calcium ionophore A23187 and treated with PS binding aptamer SIAp3, we observed striking morphological changes in RBL cells, see the microscopic images in Figure 4. RBL cells incubated with 100 µM SIAp3 showed a distinct increase in granulation as evidenced by microscopy imaging (Figure 4D), including other lower concentration-induced modest morphological changes as demonstrated in Figure 4B,C. This increased granulation can be attributed to the target-specific aptamer binding to PS in the cell membrane which is exposed as a consequence of activation with calcium ionophore A23187 and affects its distribution. It is well known that increasing calcium influx increases PS externalization thereby breaching the integrity of the cell membrane [34]. As a consequence, increased granule formation happens. PS is an important constituent of plasma membrane generally exposed during apoptosis [39], but recent findings suggest non-apoptotic PS exposure upon engagement of some receptors [32]. We assume RBL cells are in dynamic equilibrium with their surrounding environment constantly maintaining membrane turnover. Many events are responsible for the constant cycling of PS to inner and outer leaflets of plasma membranes. We think when PS-specific aptamers bind to the targets restricting the cycling mechanism, the integrity of the plasma membrane is compromised, resulting in hyper granulation and ultimately cell death.

**Figure 5:**
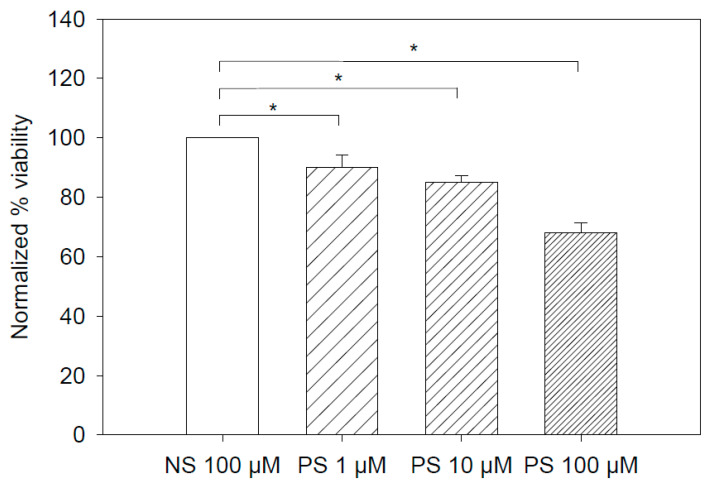
PS aptamer induces cytotoxicity in RBL cells. Calcium iohophore activated RBL cells treated with non-specific aptamer indicated as NS 100 µM (white empty column) or PS aptamer at 1, 10, and 100 µM indicated as PS 1 µM (coarse filled column), PS 10 µM (medium filled column), and PS 100 µM (fine filled column), respectively. PS were subjected to MTT assay. In our experiments, we have observed PS aptamer treated cells showed decreased cell viability. Graph represents viability values normalized to control nonspecific aptamer values. Significance was calculated using standard Students *t*-test using Sigma Plot software (SPSS Inc., Bengaluru, India). *p* value less than 0.005 was considered significant and denoted with * symbol. We used the symbol NS for SIIAp1 and PS for SIAp3 in the figure for aptamer concentrations.

Later we checked the cytotoxic effect of both SIAp3 and the control aptamer SIIAp1. With the increase in the concentration of SIAp1, RBL showed modest cytotoxicity compared to SIIAp1 (Figure 5). Monolayers of RBL cells were treated with 1 µM calcium ionophore, exposed to different concentrations (0, 1, 10, and 100 µM) of SIAp3, incubated for 24 h, and later subjected to the MTT assay [38]. In a quantitative summary on cytotoxicity results, PS aptamer treated cells showed decreased cell viability. As the PS aptamer concentration increased, we observed reduced cell viability, and due to the effects of 100 μM SIAp3, the viability was reduced by almost 35% due to the effects of 100 μM SIIAp1 (Figure 5). These comparative effects confirm the aptamer specificity in causing cytotoxicity.

## 4. Discussion

We demonstrated the PS molecule binding of PS aptamers in both liposome and cell systems. The liposome system was used to address the role of bilayer physical properties in the process of drug adsorption into the membranes. This study is crucial to assess the membrane effects of any drug as lipid membrane physical properties are found to influence the function of membrane hosted channels with versatile structural moieties [27,28,29,30,31]. In cell studies, we aimed to inspect if we also get the PS aptamers’ binding with cell surface considering especially their PS molecule binding specificity. Both fluorescence imaging and cytotoxicity data suggest positively. 

### 4.1. Lipid Bilayer Physical Properties Regulate the Binding Mechanisms of PS Aptamers with Liposomes

Amphiphiles TX100 and Cpsn are well known for their effects on lipid bilayer physical properties. Both are predicted to generally reduce the bilayer stiffness, although they are positive and negative lipid curvature profile promoters [27]. Earlier studies suggest that these amphiphiles promote the functions of various membrane-active agents in lipid bilayer membranes, e.g., the functions of membrane hosted channels of CDs [29], peptides gramicidin A [27,28,29,30,31], and alamethicin (Supplementary Materials of Reference [29]) are found to be regulated due to the amphiphiles’ effects on lipid bilayer membranes. Both of these amphiphiles increase the stability of these channels in the lipid bilayer membrane by perhaps reducing the bilayer stiffness. We, therefore, found a clear reason to check if these amphiphiles would have any effects on the membrane adsorption of aptamers. The investigated PS aptamers have been found to experience increased membrane adsorption in presence of PS in the liposome due to the effects of both amphiphiles on the membrane. Both FL measurements and DDM methods have been found to produce identical effects on the increased membrane detection of liposome adsorbed aptamers due to the effects of both TX100 and Cpsn on membranes. Like other ion channel experiments [28,29], we also observed here that TX100 is almost 3-fold more potent than Cpsn. That says, to observe identical effects on aptamer adsorption into liposomes we need a three-fold higher concentration of Cpsn over TX100 in the liposome incubating buffer. We may therefore conclude that the membrane adsorption of PS aptamers is happening in a specific PS molecule binding nature (confirmed through the PS lipid specific liposome adsorption of PS aptamers data, see Supplementary Figure S1) and that this adsorption process gets regulated due to amphiphile-induced alterations in bilayer physical properties.

### 4.2. Fluorescence Images Suggest That PS Aptamers Bind with Apoptosis-Induced Cancer Cell Surface Targets 

PS aptamers have been investigated regarding the potency of their binding with PS molecules on cell surfaces. As PS externalization is a natural process in apoptotic cells over cancerous cells experiencing no apoptosis, we decided to consider inducing apoptosis into cancer cells using colchicine to ensure having PS molecules on the cell surface. Our administered PS aptamers have been found in abundance on surfaces of apoptosis-induced cells. Comparing the captured fluorescence images Figure 3d,f on the surfaces of the (PS aptamer treated) apoptotic cells with Figure 3a,c on the surface of (PS aptamer treated) cancer (nonapoptotic) cells we understand that the cell surface migrated PS molecules experiencing substantial PS aptamer binding as the former images show the higher intensity of fluorescence over the latter ones. Quite known and well-used protein Annexin V images in Figure 3g,i are comparable with Figure 3d,f, respectively, suggesting that the source of enhanced fluorescence in Figure 3d,f is aptamer molecules that got bound to PS molecules on cell surfaces. Therefore, PS binding of PS aptamer is demonstrated undoubtedly in the biological cell environment. 

### 4.3. PS Aptamers Are Modestly Cytotoxic 

As PS aptamers have clearly been demonstrated regarding their specific PS molecule binding in in silico MD simulations [13], in vitro liposome system [6,7], and in biological cell system (current study), we planned to check on their possible cytotoxicity potency. Here, we made a judicious choice in addressing their toxicity effects on even a cancer cell system where PS migration to the cellular surface happens without inducing apoptosis, but due to the engagement of certain other agents [32]. In this study, we found PS aptamers specifically bind with PS molecules (see Figure 3) and bring changes in cell morphology (see Figure 4). RBL cell model system is not primarily aimed at this kind of study, but in our screening, we found these cells would be suitable to get activated using calcium ionophore thereby exposing the PS, because ionophore treatment induces non-apoptotic externalization of PS [32,40] thereby binding to PS aptamers on the cell surface. We think this binding prevents re-shuffling of PS and this causes loss of plasma membrane integrity. This assumption is supported by increased granularity of cytoplasm with the increase in PS aptamer concentration which can be observed as dark cytoplasm in Figure 4. 

One may raise a question whether the PS aptamer can distinctively be used as a diagnostic tool while the aptamers are also critical to cell survival, eventually causing cytotoxicity. The toxic effect of aptamers observed here is the result of cumulative effects of PS aptamer binding to PS over 12 h of incubation. Long incubation is not needed during the application of aptamers as a diagnostic tool. Moreover, cells are generally fixed before screening by flowcytometry, that is, the diagnostic test expected to be made in a controlled condition, so cytotoxicity may be avoidable during the test. Once the apoptotic program is activated, an enhanced number of PS molecules accumulate (due to lipid scrambling) on specific cell surface regions near apoptotic pores [41], so those distinguished PS-dense regions are expected to naturally attract higher number of our proposed diagnostic agents-PS aptamers. In contrast, we think when RBL cells are activated by ionophore there is continuous externalization of PS. These externalized PS molecules may attract PS aptamers randomly, and over time, this binding may result in breach of the plasma membrane integrity, leading to cell death. At this stage, we think the observed cytotoxicity is mainly because of the change in membrane properties. However, studying other specific molecular events that might get regulated due to the PS aptamer treatment would reveal further details and let us know other possible reasons that might also contribute into the triggering of cell death. This requires extensive study of several target molecules which shall be the future direction of our research.

At this stage, incubating cells with PS aptamers would serve as a good system to study their binding specificity and understand the consequences of that binding. Being a cell line of tumor origin, we believe it can also be employed as a model system for this kind of study. Results obtained from this model system not only prove the PS aptamers’ diagnostic potential but also the possibilities of their therapeutic applications. Further experiments screening the effect of PS aptamers employing well-established cancer cell lines of different tissues will be more useful, and also, studying the effect of PS aptamers in in vivo tumor models will certainly enhance our findings. We are actively planning to extend our studies to cover these additional assays.

## 5. Conclusions

PS aptamers have earlier been designed theoretically, validated for their target PS molecule binding potency in in silico MD simulations and in vitro liposome system experiments. The PS binding of PS aptamers has been molecularly confirmed energetically considering their charge-based electrostatic and van der Waals interactions [13]. In the current study, we wished to extend our understanding of this specific aptamer-target lipid binding potency in biological cell systems. Firstly, we mimicked the liposome experiments [6,7] to address an important molecular mechanism of whether membrane physical properties might influence the liposome adsorption process. It is found that two amphiphiles, TX100 and Cpsn, both known to reduce the membrane stiffness, enhance the liposome adsorption of PS aptamers. As usual, the control aptamer’s binding to liposome was not found to be influenced due to either amphiphile. This membrane physical property-induced upregulation of the membrane adsorption of aptamers appears qualitatively in line with other membrane-active agents, e.g., CD agent colchicine [29] and peptides gramicidin A and alamethicin [27,28,29,30,31], all of which experience enhanced pore stability inside lipid bilayer membranes due to these amphiphiles’ membrane effects. Fluorescence imaging demonstrates clearly that PS aptamers bind with PS molecules available on the surface of the apoptotic cells. As expected, the surface of cancer cells without apoptosis being induced fails to show the presence of PS molecules, so naturally, negligible fluorescence was detected there, suggesting no PS aptamer binding on the cell surface. These imaging data suggest that PS aptamers may be utilized as PS externalization detection kits in apoptotic cells. The cytotoxicity assays demonstrate that PS aptamers, compared to nonspecific aptamers, are modestly cytotoxic. Here we used a cell line where we induced nonapoptotic PS externalization, so again the cytotoxicity we observed was perhaps due to the mechanism of the specific PS molecule binding of PS aptamers, thus slowly breaching plasma membrane integrity. Results in this article will extend our understanding of the aptamer binding to target biomolecules in cellular systems and help us plan for designing and utilizing aptamers as novel molecular agents in drug discovery. The use of PS aptamers to diagnose the induction of apoptosis during chemotherapy applications in cancer treatment may appear like a realistic possibility.

## Data Availability

Not applicable.

## Supplementary Materials

The following are available online at https://www.mdpi.com/article/10.3390/membranes12010037/s1, Figure S1: Affinity of liposome binding and selectivity for DNA aptamers. FL was measured in the ‘relative fluorescence unit (RFU)’ for various concentrations of aptamers. SIAp1, 3, 4 and SIIAp1, 2 are presented in Table S1. Quantitative difference in liposome binding is clearly seen among different aptamer sequences. Taken from ref. [7]. Table S1: The two sets of DNA aptamers designed for lipid binding [6,7]. The third and fourth column list probabilities of designed aptamers and phospholipid within their mutual distance 6–16 Å (for details see [6,7]). SI is designed based on total energy and SII is designed using interaction energy.
