# Supplementary material for: Lipid Specific Membrane Interaction of Aptamers and Cytotoxicity
Source: Membranes (Basel). 2021 Dec 27;12(1):37. doi: 10.3390/membranes12010037 (PMC8780203; doi:10.3390/membranes12010037)
Supplement: Supplementary file 1 [file membranes-12-00037-s001.zip › membranes-1505381-supplementary.pdf]

membranes

Supplementary Materials

Supplementary Materials: Lipid Specific Membrane Interaction of Aptamers and Cytotoxicity

Md. Ashrafuzzaman [1,*], Hanouf A. M. AlMansour [1], Maha A. S. AlOtaibi [1], Zahid Khan [1], and Gouse M. Shaik [1]

[1] Biochemistry Department, Science College, King Saud University, Riyadh 11451, Saudi Arabia
* Correspondence: mashrafuzzaman@ksu.edu.sa

Table S1. The two sets of DNA aptamers designed for lipid binding [6,7]. The third and fourth column list probabilities of designed aptamers and phospholipid within their mutual distance 6–16Å (for details see [6,7]). SI is designed based on total energy and SII is designed using interaction energy.

Name	Sequence (5' → 3')	PS	PC
SIAp1	AAAAGA	0.23 ± 0.11	0.06 ± 0.02
SIAp2	AAAGAG	N/A	N/A
SIAp 3	TAAAGA	0.57 ± 0.15	0.31 ± 0.15
SIAp 4	AAAGAC	0.54 ± 0.19	0.41 ± 0.08
SIIAp1	CAGAAAAAAAC	0.4 ± 0.2	0.41 ± 0.14
SIIAp2	CAGAAAAAAAT	0.31 ± 0.12	0.29 ± 0.19

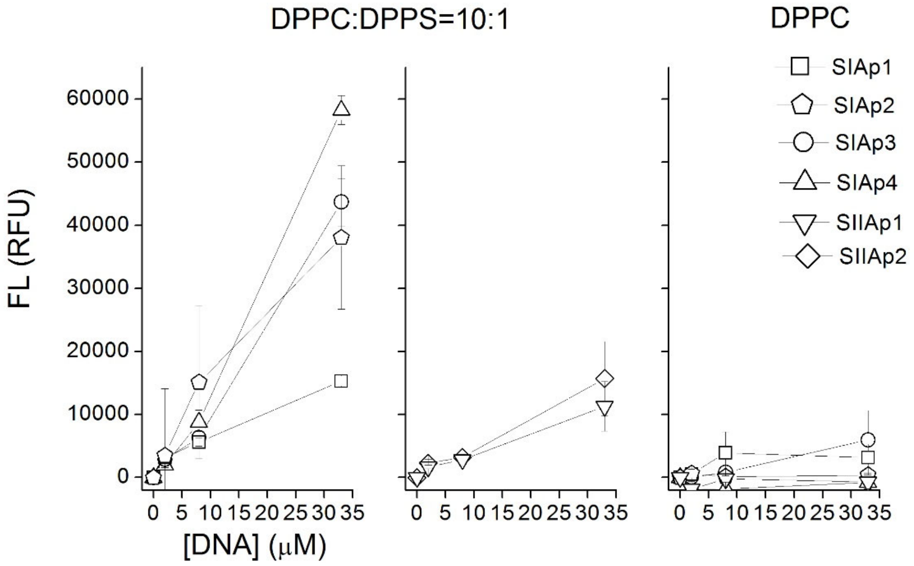

Figure S1. Affinity of liposome binding and selectivity for DNA aptamers. FL was measured in the 'relative fluorescence unit (RFU)' for various concentrations of aptamers. SIAp1, 3, 4 and SIIAp1, 2 are presented in Table 1. Quantitative difference in liposome binding is clearly seen among different aptamer sequences. Taken from ref. [7].

Aptamer-Liposome Binding Assays

The liposomes were prepared with DPPC, DPPS, and cholesterol (all in powder form) obtained from Avanti Polar Lipids (Alabaster, AL, USA). They were used without further purification. DNA designed aptamer sequences containing the 6-carboxyfluorescein (6-FAM) fluorescent tag attached at the 3' end of the DNA sequence were obtained from Integrated DNA Technologies (Coralville IA, USA). 96 well assay plates for fluorescence assays using FLUOstar OPTIMA (BMG LABTECH GmbH, Offenburg, Germany) were from Corning Inc. (New York, USA). There were eight replicates of samples prepared for each aptamer concentration in the studies. A 2:1 ratio of PC to cholesterol was prepared by dissolving 100 mg of DPPC and 24.45 mg of cholesterol in 10 mL of chloroform to prepare control liposomes that do not contain PS. PS containing liposomes contained a combination of DPPC and DPPS in a 10 to 1 molar ratio.

Regarding the protocol of binding assays, we encourage readers to consult refs. [6,7]. 10 mL of a chloroform solution of the phospholipids and cholesterol was evaporated to dryness by gently blowing nitrogen gas into the solution in a test tube. The dried lipid film created on the walls of the test tube was kept overnight to remove remnants of chloroform. 10 mL of HEPES buffer was then added to the dried lipid film and the mixture was vortexed at room temperature (~ 25 degree C) to create lipid vesicles (primarily multilamellar) in buffer (e.g., see Hope et al., 1986). 1 mL of the liposome formulation in HEPES buffer was transferred to each Eppendorf tube followed by addition of appropriate volume of the test fluorescent aptamer as a solution in Tris/EDTA buffer (1 mM stock or stock with further dilution) to ensure the desired experimental aptamer concentration in solution.

The aptamer/phospholipid mixture was incubated for 40 minutes in the dark. We then centrifuged the eppendorf tubes to pellet lipid and the bound aptamers at 25 degree C using Hettich Rotina 35R (Het-tich America LLP, Buford, GA, USA) at 19,520 g for 1 min. Supernatants (buffer and unbound aptamers) were then removed. 1 mL of HEPES buffer was added to the eppendorf tubes to resuspend the phospholipid followed by centrifugation and the supernatants were again removed. This process was repeated three times to effectively remove unbound aptamers. The required amount of HEPES buffer was then added to each eppendorf tube containing bound aptamer/phospholipids to make the final volume 1 mL. 100 μL from this final suspension was loaded onto a 96 well plate and the fluorescence intensity was measured for each sample. PC software version V1.30 R4 (BMG LABTECH GmbH, Offenburg, Germany) was used for data collection. Excitation filter was at 485 nm and emission filter at 520 nm.
